# Personality traits in musicians with different types of music performance anxiety

**DOI:** 10.3389/fpsyg.2024.1398095

**Published:** 2024-08-16

**Authors:** Claudia Spahn, Franziska Krampe-Heni, Jesper Hohagen, Anna Immerz, Manfred Nusseck

**Affiliations:** ^1^Freiburg Institute for Musicians’ Medicine, University of Music Freiburg, Freiburg University Medical Center, Faculty of Medicine, University of Freiburg, Freiburg Center for Music Research and Teaching, Freiburg, Germany; ^2^Department of Pediatrics, Childrens Hospital Munich Schwabing and Harlaching, TUM School of Medicine, Kölner Platz, Munich, Germany; ^3^Kinderzentrum kbo Munich, Heiglhofstraße, Munich, Germany

**Keywords:** music performance anxiety, personality traits, neuroticism, amateur musicians, professional orchestra musicians

## Abstract

**Introduction:**

This study investigated the relationship between personality traits and MPA in the course of a specific performance.

**Methods:**

For this purpose, symptoms of MPA, functional coping with MPA and performance-related self-efficacy of a sample of 393 musicians including 23% professional, 49% non-professional orchestra musicians and 28% singers in amateur choirs were categorized and then used to analyze differences in the personality traits. The questionnaires used were the NEO-FFI and the PQM.

**Results:**

The results showed that professional orchestra musicians had significantly higher openness and conscientiousness than non-professional orchestra musicians and amateur choir singers. Musicians who had few symptoms of MPA, positive coping with MPA and high self-efficacy across a specific performance (Type 1) have low neuroticism in their personality traits. Regarding MPA, the personality traits were correlated with all MPA scales after the performance but less with MPA scales before and during the performance.

**Discussion:**

Results could indicate that personality traits play a particularly important role in the processing of performance experiences after the performance and suggest focusing on the situation after the performance in particular with professional orchestra musicians.

## 1 Introduction

A variety of factors contributes to the experience of music performance anxiety (MPA). MPA is generally described as a state of excitement, which can cause negative symptoms of stress reactions (Spahn, 2015; Guyon et al., 2020), but can at its optimal level also enhance a performance. These factors cover a wide spectrum of personal characteristics and self-perceptions, such as self-efficacy, emotionality, perfectionism, fear of negative evaluation, and manifestations of general anxieties (Kenny, 2011; Spahn, 2015; Osborne and Kirsner, 2022). The personality of musicians in particular is an important factor in the development of MPA (Smith and Rickard, 2004; Chattin, 2019; Rodriguez-Mora and Lopez Diaz, 2020; Herman and Clark, 2023).

To describe personality traits, the multidimensional personality inventory NEO-FFI (1989) developed by Costa and McCrae (1989) was mostly used, with which five essential characteristics, the so-called Big Five, are surveyed according to the five-factor model. These factors are (1) openness to experience (2) conscientiousness, (3) extraversion (4) agreeableness and (5) neuroticism.

*Openness* to experience stands for tolerance and interest towards new things (also on an intellectual level), and a positive attitude towards art and culture. Open people are usually inventive, innovative, decent, level-headed and interested (Pervin, 2000). *Conscientiousness* stands for the realization of the goal direction of action. People with a pronounced conscientiousness are well-planned, conscientious, reliable, action-oriented and ambitious (Pervin, 2000). *Extraversion* stands for the passion with which emotions are lived out, on the one hand in contact with one’s environment, and on the other hand one’s own impulses, such as the expression of joy. Extraverted people are typically talkative, active, powerful, decisive and bold. Introverts are accordingly afflicted with the opposite characteristics (Pervin, 2000). *Agreeableness* stands for helpfulness and selflessness. People with this trait are confident, gentle, collaborative, generous and giving (Pervin, 2000). *Neuroticism* stands for the assimilation and handling of one’s own emotionality to external influences. Neurotic people are often impatient, restless, frustrated, sensitive and fickle (Pervin, 2000).

Using the NEO-FFI personality trait questionnaire, a number of studies have investigated the influence of musicians’ personality traits on the general manifestation of MPA. Rodriguez-Mora and Lopez Diaz (2020) investigated a sample of 72 musicians between 16 and 54 years old with the KMPAI-E (Kenny, 2011) to measure MPA and with the NEO-FFI to measure personality traits. The results showed a positive correlation between MPA and neuroticism (*p* < 0.01) and a negative correlation of MPA with extraversion (*p* < 0.01) and responsibility (*p* < 0.05).

In line with this, Chattin (2019) found in her study with 55 collegiate music piano majors a significant relationship between the Big Five personality types and levels of MPA. Overall results indicated that certain personality constructs studied in this research do have an impact on MPA. Findings suggest that neuroticism, worry, self-focus and somatic tension affect performers negatively, while openness, conscientiousness, and perceived control likely helped to alleviate MPA. Males reported higher levels of perceived control than females.

Herman and Clark (2023) conclude in their review on MPA that studies consistently report significant associations between MPA and high levels of maladaptive personality traits, neuroticism and MPA being strongly associated (Valentine et al., 1995; Langendörfer et al., 2006; Thomas and Nettelbeck, 2014).

More recently, studies have been conducted to determine whether different types can be described in the way MPA occurs (Papageorgi, 2021; Spahn et al., 2021). Papageorgi (2021) identified three clusters among adolescent instrumental learners with low, medium and high anxiety, who developed adaptive MPA through good self-esteem and positive coping as well as maladaptive MPA in the case of negative self-perceptions.

Spahn et al. (2021) investigated how dispositional MPA relates to performance-specific MPA. Regarding performance-specific MPA, they found three different MPA types across a specific performance. Type 1 describes musicians who have few symptoms of MPA throughout the performance, show functional coping with MPA, and have a stable and well-developed self-efficacy. Type 2 describes musicians who begin their performance with rather high symptoms of MPA but can positively reduce these by the end of the performance and show high values in self-efficacy and in functional coping. Type 3 contains musicians who begin their performance with some symptoms of MPA, which increase to the end of the performance. The values of self-efficacy and functional coping in this type are rather low. Professional musicians were more often distributed in MPA Type 3 than amateur musicians.

The discrimination of the three types of MPA was also found in young amateur brass musicians (Spahn et al., 2023). Most of the musicians were found to be in MPA Type 1. These findings were in accordance with the three clusters found by Papageorgi (2021) by using similar criteria. This shows the reliability of the classification of three MPA types.

The present study aimed to examine for the first time whether musicians in the different performance-specific MPA groups (Spahn et al., 2021) differ in terms of their personality traits.

The objective was to investigate the relationship between personality traits and MPA in the course of a specific performance:

How do personality traits relate with symptoms of MPA, functional coping with MPA and performance-related self-efficacy before a performance, during the performance and after the performance?

For this purpose, symptoms of MPA, functional coping with MPA and performance-related self-efficacy of a sample of musicians were categorized and then used to analyze differences in the personality traits.

## 2 Material and methods

### 2.1 Participants

The sample of this study consisted of 393 musicians including 23% professional and 49% non-professional orchestra musicians and 28% singers in amateur choirs (see Table 1). The classification into these musical subgroups was based on the musical level of the orchestras. Professional orchestra musicians had main occupations at radio symphony orchestras or philharmonic orchestras. The non-professional orchestra musicians came from student orchestras and semiprofessional orchestras. The amateur choir singers were amateur singers in semiprofessional choirs. The sample is a sub-sample of the study Spahn et al. (2021).

**Table 1:** Characteristics of the sample.

	Professional orchestra musicians	Non-professional orchestra musicians	Amateur choir singers
Amount (*N* = 393)	*N* = 89 (23%)	*N* = 195 (49%)	*N* = 109 (28%)
Gender (% female)	43.2%	56.4%	79.2%
Age (in years, SD)	43.4 (13.2)	25.2 (9.6)	28.7 (13.4)

In total, 59.6% were female musicians. There was a significant distribution difference between the musical subgroups [*χ*^2^ (398, 2) = 27.7, *p* < 0.001] with more females among the choir singers than among the orchestra musicians.

The mean age of the sample was 30.2 years [SD = 13.6 years]. The professional orchestra musicians were significantly older than the non-professional orchestra musicians and the amateur choir singers [*F* (2,381) = 74.5, *p* < 0.001], with the latter two being mostly students.

The distribution of the instruments were 45% strings (with 79% in the professional and 57% in the non-professional orchestras), 13% woodwind instruments (with 12% in the professional and 22% in the non-professional orchestras), 29% singers (all in the choirs) and 13% other instruments such as percussions and brass instruments.

### 2.2 Measures

#### 2.2.1 NEO-five-factor-inventory

For measuring the personality traits, the German version of the NEO-FFI (NEO-Five-Factor-Inventory) was used (Körner et al., 2002). The questionnaire measures Neuroticism, Extraversion, Openness, Agreeableness and Conscientiousness. In the short version with 30 items (Körner et al., 2008) statements regarding the described personality characteristics were rated on a Likert scale from 0 (strongly disagree) to 4 (strongly agree) and a mean scale value was calculated for each personality trait.

#### 2.2.2 Performance-specific questionnaire for musicians

MPA was measured with the Performance-specific Questionnaire for Musicians (PQM, see Spahn et al., 2021). The questionnaire refers to the times directly before, during and after a music performance. It needs to be completed shortly after a performance, whereby the questions relating to the times before and during the performance are answered retrospectively. In total, 33 items need to be answered on a Likert scale between 1 (does not apply) and 5 (fully applies) with 11 items per time point. At each time point, three scales were calculated: functional coping, referring to the positive effects of MPA, symptoms of MPA, referring to physical and mental impacts of MPA, and self-efficacy, which comprises the belief in the personal ability to perform.

With the responses of 532 musicians, three types of MPA have been categorized using a cluster analysis (Spahn et al., 2021). Musicians in Type 1 reported of few symptoms of MPA, showed high functional coping with MPA, and a stable and well-developed self-efficacy across the whole performance. Musicians of Type 2 began the performance with considerable high symptoms of MPA but reduced them by the end of the performance. They also showed high values in self-efficacy and functional coping. Type 3 contained musicians with moderate symptoms of MPA at the beginning of the performance, which increased to the end of the performance. They showed low values in self-efficacy and functional coping. The musicians were classified 50% as Type 1, 27% as Type 2 and 23% as Type 3 (Spahn et al., 2021).

A further seven items were used to assess the self-rated quality of the performance. The items refer to specific musical parameters, such as tempo, intonation, dynamics, and were rated on a Likert scale from 1 (badly) to 6 (excellent). The mean value of all seven items builds a specific judgment score. The higher the score the better the musicians valued their own performance quality.

### 2.3 Design

After concerts of the orchestras and choirs, the musicians were asked to fill in the questionnaires as pen-and-paper version. The concerts were regular concerts of the music group in line with their normal performance schedule and with standard repertoire. In the off-stage facilities, the musicians were given the questionnaire by an experimenter, who informed them about the study and that participation was completely anonymous and voluntary. The study was granted ethical approval by the Ethics Committee of the University Clinic Freiburg.

### 2.4 Data analysis

The statistical analyses were performed using SPSS (Version 29, Armonk, NY: IBM Corp.). Descriptive statistics including mean and standard deviation (SD) were calculated for each variable. To analyse distribution differences of non-parametric variables Chi-square (*χ*^2^) tests were performed. Parametric comparisons on the PQM scales and the NEO-FFI personality traits were calculated with multivariate ANOVAs. On significance, *post hoc* analyses with Tukey HSD correction were performed. A canonical correlation analysis (CCA) was conducted using the NEO-FFI personality traits and the PQM scales to evaluate the multivariate shared relationship between the two variable sets. The level of statistical significance was set at 0.05.

## 3 Results

### 3.1 NEO-five-factor-inventory

The multivariate analysis of the NEO-FFI scales across the three musical subgroups within the whole sample found significant effects in the scales openness [*F* (2,390) = 3.38, *p* = 0.035] and conscientiousness [*F* (2,390) = 3.83, *p* = 0.022] (Table 2). The effect in openness was caused by higher values in the professional musicians compared to the choir singers [Post-Hoc, *p* = 0.035] and in conscientiousness by higher values in the professional musicians compared to the amateur musicians [Post-Hoc, *p* = 0.016].

**Table 2:** Mean values of the NEO-FFI scales with standard deviation by musical subgroup (*p < 0.05).

NEO-FFI	Professional orchestra musicians (*N* = 89)	Non-professional orchestra musicians (*N* = 195)	Amateur choir singers (*N* = 109)
Neuroticism	1.38 (0.70)	1.51 (0.80)	1.38 (0.77)
Extraversion	2.52 (0.50)	2.60 (0.60)	2.54 (0.55)
Openness	2.92* (0.65)	2.74* (0.68)	2.68* (0.66)
Agreeableness	2.99 (0.58)	2.83 (0.70)	2.91 (0.72)
Conscientiousness	3.05* (0.54)	2.82* (0.66)	2.90* (0.71)

There were also significant main effects for gender in the neuroticism scale [*F* (1,383) = 6.32, *p* = 0.001] and in the agreeableness scale [*F* (1,383) = 15.66, *p* < 0.001] with higher values for the female musicians in both scales. However, there were no significant interaction effects for gender and musical subgroup.

### 3.2 Performance-specific questionnaire for musicians

The mean values of the PQM scales for each musical subgroup are listed in Table 3. The multivariate analysis across the three musical subgroups found several significant main effects. Before the performance, in the scale symptoms of MPA [*F* (2,390) = 6.24, *p* = 0.002] the non-professional musicians were significantly higher than the professional musicians [Post-Hoc, *p* = 0.037] and the choir singers [Post-Hoc, *p* = 0.004] and in the self-efficacy scale [*F* (2,390) = 5.11, *p* = 0.006] the choir singers were significantly higher than the professional [Post-Hoc, *p* = 0.013] and the non-professional orchestra musicians [Post-Hoc, *p* = 0.016]. During the performance, in the scale functional coping [*F* (2,390) = 2.19, *p* = 0.02] the choir singers were significantly higher than the non-professional musicians [Post-Hoc, *p* = 0.016], in the scale symptoms of MPA [*F* (2,390) = 10.68, *p* < 0.001] the non-professional musicians were significantly higher than the choir singers [Post-Hoc, *p* < 0.001] and the professional musicians [Post-Hoc, *p* = 0.013] and in the self-efficacy scale [*F* (2,390) = 5.98, *p* = 0.003] the choir singers were significantly higher than the professional [Post-Hoc, *p* = 0.006] and the non-professional orchestra musicians [Post-Hoc, *p* = 0.008]. After the performance, in the scale functional coping [*F* (2,390) = 15.91, *p* < 0.001] the choir singers were significantly higher than the professional [Post-Hoc, *p* < 0.001] and the non-professional orchestra musicians [Post-Hoc, *p* < 0.001] and in the scale symptoms of MPA [*F* (2,390) = 7.83, *p* < 0.001] the choir singers were significantly lower than the professional [Post-Hoc, *p* < 0.001] and the non-professional orchestra musicians [Post-Hoc, *p* = 0.003].

**Table 3:** Mean values of the PQM scales with standard deviation by musical subgroup (*p < 0.05 and **p < 0.01; in bold: highest value if significant).

PQM	Professional orchestra musicians	Non-professional orchestra musicians	Amateur choir singers
Before the performance			
Functional coping	4.21 (0.68)	4.19 (0.75)	4.29 (0.73)
Symptoms of MPA	1.92 (0.94)**	2.20 (0.91)**	1.86 (0.78)**
Self-efficacy	3.79 (0.71)**	3.84 (0.69)**	4.07 (0.71)**
During the performance			
Functional coping	4.21 (0.82)*	4.16 (0.70)*	4.41 (0.75)*
Symptoms of MPA	1.76 (0.83)**	2.05 (0.79)**	1.64 (0.72)**
Self-efficacy	3.98 (0.71)**	4.04 (0.67)**	4.28 (0.69)**
After the performance			
Functional coping	4.06 (0.71)**	4.30 (0.73)**	4.60 (0.57)**
Symptoms of MPA	1.77 (0.76)**	1.68 (0.70)**	1.40 (0.72)**
Self-efficacy	3.98 (0.81)	4.02 (0.81)	4.20 (0.84)

### 3.3 Correlations between NEO-five-factor-inventory and performance-specific questionnaire for musicians

**Figure 1:**
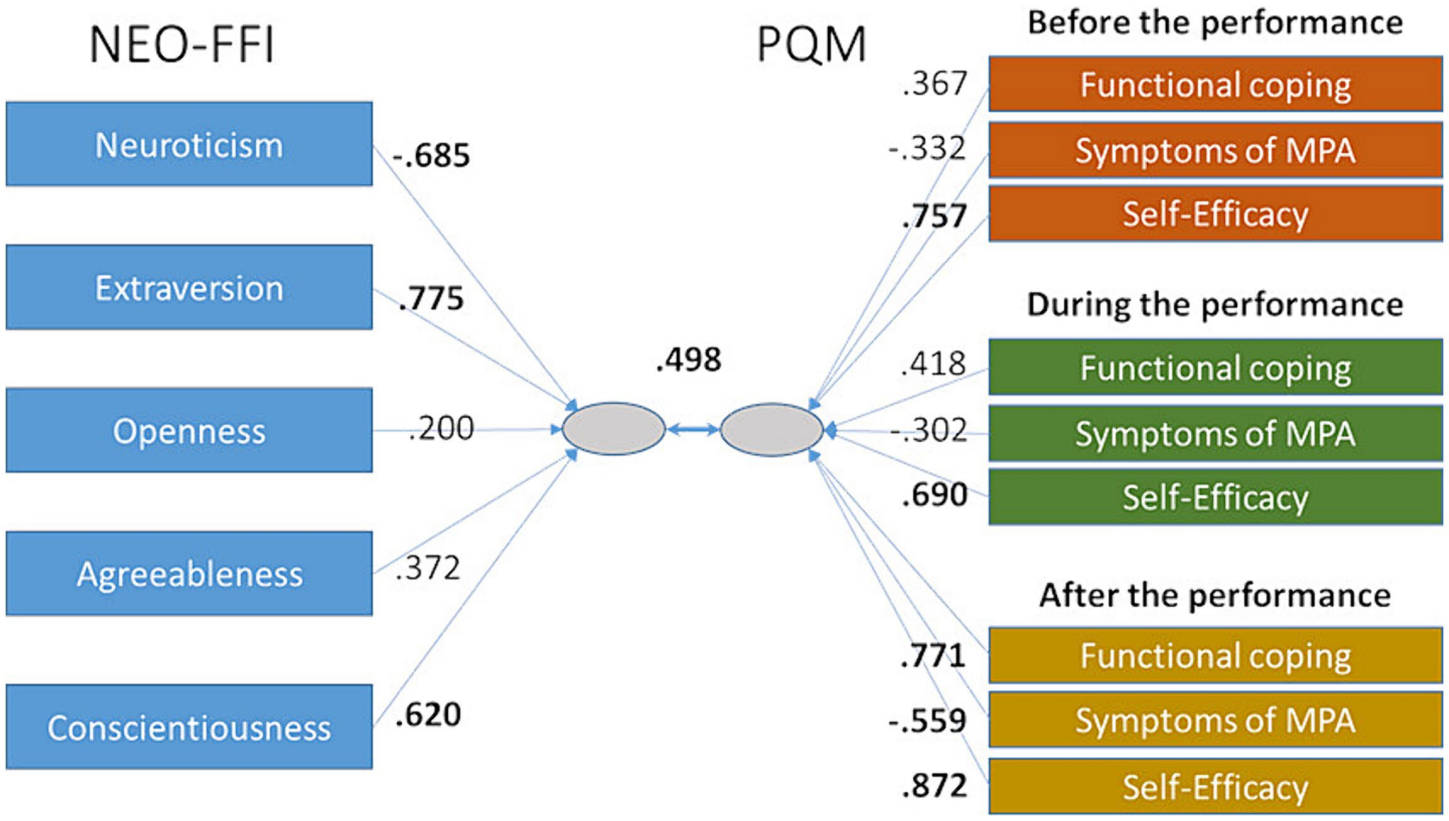
Outcome of the CCA between NEO-FFI and PQM showing the canonical correlation coefficients (in bold: relevant correlations).

The canonical correlation analysis (CCA) yielded five functions with 59.5% explained variance for the full model across all functions with the first three functions being significant. Since the first function [*F* (45,1,698,5) = 4.81, *p* < 0.001] already explained 55.4% of the variance shared between the variable sets, only this function was considered for further analysis. In this function, the questionnaires correlated with Pearson’s *r* = 0.498 with each other (see Figure 1). The explained variance for the NEO-FFI questionnaire was 32.7% and for the PQM 35.9%.

For the analysis of the individual predictor variables, only correlation coefficients above *r* = 0.5 were considered. The results showed that neuroticism, extraversion and conscientiousness were the primary contributors for the prediction on the NEO-FFI side and the self-efficacy scales at all performance times as well as all scales after the performance on the PQM side. While extraversion and conscientiousness were positively related, neuroticism was negatively correlated. For the PQM scales, self-efficacy and functional coping showed positive and symptoms of MPA negative correlation coefficients.

The correlations between the NEO-FFI personality traits with the self-assessed judgment score of the performance quality found significant correlation coefficients only for neuroticism [*r* = −0.22, *p* < 0.01] and extraversion [*r* = 0.23, *p* < 0.01]. These correlations are moderate to low. The negative correlation of neuroticism describes that musicians with higher neuroticism rated their performance lower. Musicians with higher values of extraversion rated their performance as better.

### 3.4 Types of MPA

**Figure 2:**
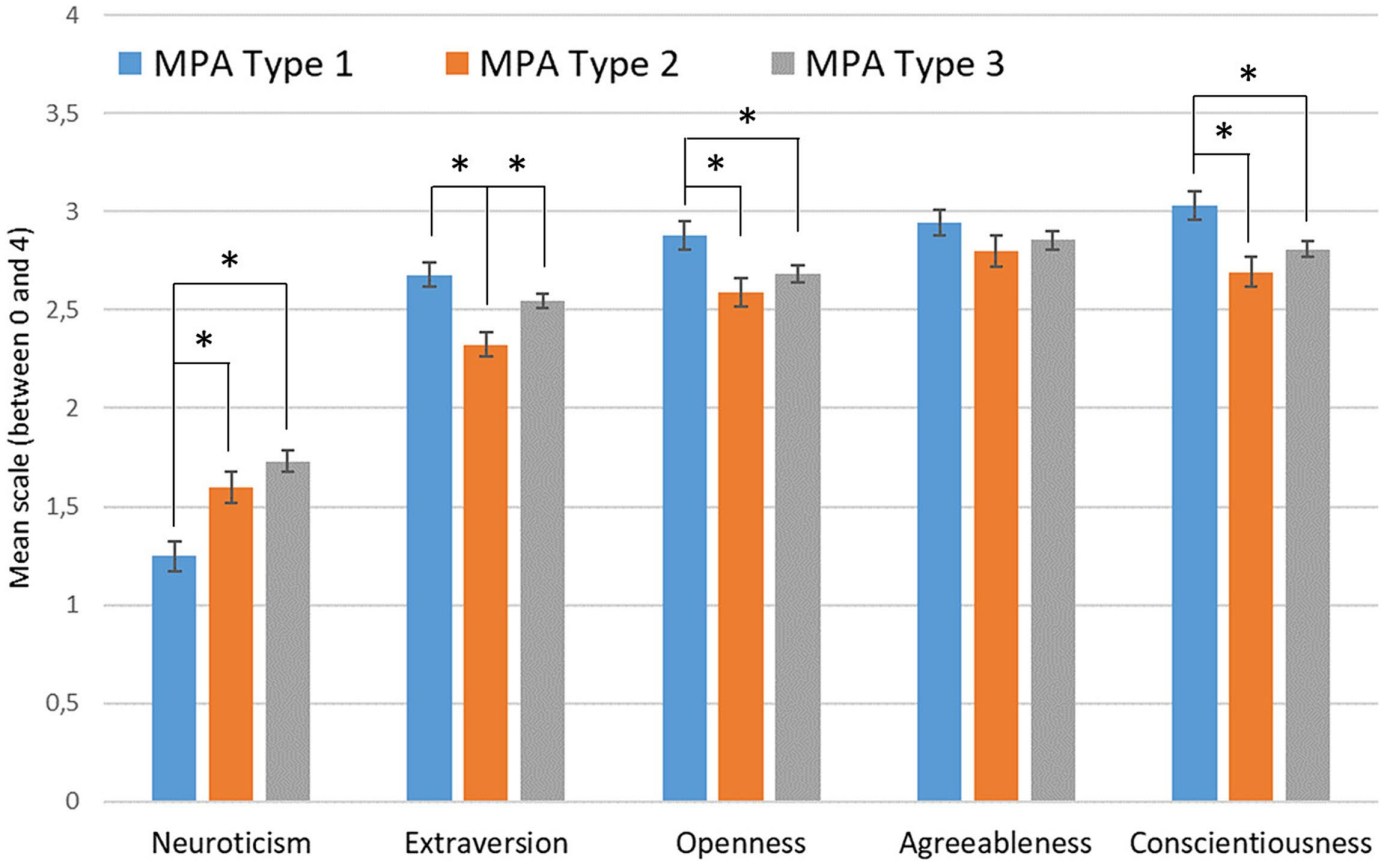
Mean values of the NEO-FFI scales (error bars show standard error of the mean) by MPA Type (*p < 0.05).

The types of MPA were distributed to 52% in Type 1, 23% in Type 2 and 24% in Type 3. In a multivariate analysis, extraversion, openness, neuroticism and conscientiousness showed significant main effects across the three types of MPA [*F* (2,390) > 7.0, *p* < 0.01] (see Figure 2).

The detailed comparisons showed that musicians of MPA Type 1 had significantly lower mean values in the neuroticism trait than the musicians of the other Types [Post-Hoc, *p* < 0.01]. Furthermore, the musicians of MPA Type 1 had also significantly higher values in the extraversion scale than the musicians of Type 2 [Post-Hoc, *p* < 0.01] but not of Type 3. Here, the musicians of Type 2 had significantly lower values than of Type 3 [Post-Hoc, *p* = 0.017]. For the openness, musicians in Type 1 were significantly higher compared to musicians in Type 2 [Post-Hoc, *p* = 0.002] and in Type 3 [Post-Hoc, *p* = 0.047]. Musicians of the MPA Type 1 were also higher in the conscientiousness trait than musicians of Type 2 [Post-Hoc, *p* < 0.01] and of Type 3 [Post-Hoc, *p* = 0.015]. Except for the extraversions scale, the mean values of the musicians in Type 2 and 3 did not differ significantly in the personality traits.

## 4 Discussion

This study investigated the relationship between personality traits and performance-related MPA in professional and non-professional orchestra musicians and amateur choir singers.

### 4.1 Personality traits

In the NEO-FFI personality traits, the three musical subgroups differed only in openness and conscientiousness. Professional orchestra musicians showed significantly higher openness and conscientiousness than non-professional orchestra musicians and amateur choir singers.

This is in contrast to the results of Kuckelkorn et al. (2021), where the other personality traits did also differ significantly between professional and non-professional musicians. Using the NEO-FFI personality trait questionnaire, Kuckelkorn et al. (2021) found differences between professional and amateur musicians with higher values in openness and neuroticism as well as lower values in agreeability and conscientiousness in professional musicians compared to the amateur musicians.

Our results appear comprehensible due to the different situation and requirements in which professional musicians find themselves compared to non-professional musicians and choir singers. For example, professional musicians must be more conscientious in order to meet professional standards. At the same time, being open to new experiences is particularly well suited to the profession of orchestral musician, especially as a high degree of flexibility is required in the music profession.

Female musicians in our study showed higher values in neuroticism and agreeableness than male musicians. This was found similarly in other studies (Herman and Clark, 2023).

### 4.2 Performance-related MPA

In the evaluation of the PQM scales, the amateur choir singers stood out in comparison to professional and non-professional orchestra musicians with the highest self-efficacy before and during the performance. Amateur choir singers were best able to cope with MPA during the performance and had the fewest symptoms of MPA after the performance. Ryan and Andrews (2009) were also able to show that choir singers suffer from MPA, but that performances in instrumental ensembles triggered stronger MPA than choir performances.

Possibly due to less experience and playing skills than the professional orchestra musicians, the non-professional orchestra musicians showed the strongest symptoms of MPA before and during the performance. Interestingly, the professional orchestra musicians had the lowest self-efficacy at all three time points of the performance and remained at the same level of symptoms of MPA, especially after the performance. As self-efficacy can be influenced by high expectations and perfectionism, these lower values among professional orchestra musicians can be linked to this. From the perspective of musicians’ medicine, it could be important for professional orchestra musicians to consciously relax in the situation after the concert and improve their self-efficacy through positive self-assessment.

### 4.3 Correlation between personality traits and MPA

The results of our study show a positive correlation between self-efficacy and extraversion in relationship to MPA. These results were generally supportive of the theoretically expected relationships between personality traits and MPA in particular and are in accordance with Rodriguez-Mora and Lopez Diaz (2020).

Similarly, the findings on the correlation of higher levels of neuroticism and stronger MPA in other studies (Valentine et al., 1995; Smith and Rickard, 2004; Langendörfer et al., 2006; Thomas and Nettelbeck, 2014; Chattin, 2019) were confirmed. Additionally, musicians with higher neuroticism rated their performance lower. Musicians with higher values of extraversion rated their performance as better.

The relation of personality traits and aspects of MPA at a performance differed between amateur and professional musicians. Interestingly, among the professional musicians, neuroticism was only associated with the post-performance situation. This could indicate that personality traits play a particularly important role in the processing of performance experiences after the performance and would again focus in particular on the situation after the performance with professional orchestra musicians.

### 4.4 Types of performance-specific MPA and personality traits

The musicians were divided into three MPA types according to Spahn et al. (2021) and in accordance with Spahn et al. (2023) and Papageorgi (2021). Musicians of Type 1 differed significantly from musicians of the other two Types 2 and/or 3 in the personality traits by higher extraversion, openness and conscientiousness and lower values for neuroticism.

In particular, Type 1 differed from the other two types in that openness and conscientiousness were stronger here. Chattin (2019) also found both personality traits to be able to reduce MPA. This could be interpreted to the effect that these musicians have more flexibility in the performance situation while at the same time being more conscientious in their preparation.

The correlations between low neuroticism and adaptive MPA from previous studies (Smith and Rickard, 2004; Chattin, 2019; Rodriguez-Mora and Lopez Diaz, 2020; Herman and Clark, 2023) are thus also confirmed with regard to a specific performance situation. The proximity between anxiety and neuroticism also makes it possible to relate the present results to the study results of Papageorgi (2021).

### 4.5 Limitations of the study

Even though the present study is based on a large sample, it should be borne in mind that the sample is rather heterogeneous, consisting of professional and non-professional musicians, as well as orchestra musicians and choir singers. The results obtained here should therefore be replicated in large, homogeneous samples.

Furthermore, the PQM was applied for each musician only once and for one performance. It might be interesting to investigate how personality traits may affect more than one performance of the same person.

## 5 Conclusion

The present study shows that musicians who have few symptoms of MPA, a positive coping with MPA and a high self-efficacy regarding performance have lower neuroticism and higher openness and conscientiousness in their personality traits. This indicates an influence of personality traits on the performance practice of musicians. The results confirm the findings of previous studies and emphasize the importance of identifying musicians who are less advantaged by their personality traits and providing them with long-term support in the form of appropriate techniques and measures to improve their self-efficacy and coping with MPA.

## Data availability statement

The raw data supporting the conclusions of this article will be made available by the authors, without undue reservation.

## Ethics statement

The studies involving humans were approved by Ethics Committee of the University Clinic Freiburg. The studies were conducted in accordance with the local legislation and institutional requirements. Written informed consent for participation was not required from the participants or the participants’ legal guardians/next of kin because the study was completely anonym and the participants read a study information with all necessary data protection information. With filling in the questionnaire, they agreed for participation. The study was reviewed and approved by Ethics Committee of the University Clinic Freiburg.

## Author contributions

CS: Conceptualization, Methodology, Project administration, Supervision, Writing – original draft, Writing – review & editing. FK-H: Data curation, Methodology, Writing – review & editing. JH: Conceptualization, Formal analysis, Writing – review & editing. AI: Conceptualization, Methodology, Writing – review & editing. MN: Conceptualization, Formal analysis, Methodology, Writing – review & editing.
