# Supplementary material for: Personality traits in musicians with different types of music performance anxiety
Source: Front Psychol. 2024 Aug 16;15:1398095. doi: 10.3389/fpsyg.2024.1398095 (PMC11362056; doi:10.3389/fpsyg.2024.1398095)
Supplement: Supplementary file 1 [file Data_Sheet_1.PDF]

PQM (Performance-specific Questionnaire for Musicians v4)
(Spahn & Nusseck, 2016)

Please address the following statements to the performance you have just completed. Read the full sentence through before choosing the answer, which most closely fits.

Please do not linger too long on each sentence. There are no right or wrong answers! Please cross only one box per statement.

A few minutes before the performance...

		1 (Not true at all)	2	3	4	5 (Very true)
**1**	… I could concentrate on the work at hand.	☐	☐	☐	☐	☐
**2**	… I felt inner strength for the performance I was about to do.	☐	☐	☐	☐	☐
**3**	… I could sense signs of agitation in my body.	☐	☐	☐	☐	☐
**4**	… I managed to control my agitation and stay calm.	☐	☐	☐	☐	☐
**5**	… I could imagine the audience enjoying my performance.	☐	☐	☐	☐	☐
**6**	… I felt limited or disabled due to the way my body reacted to my agitation.	☐	☐	☐	☐	☐
**7**	… I was looking forward to going on stage and showing what I could do.	☐	☐	☐	☐	☐
**8**	… I could control my agitation.	☐	☐	☐	☐	☐
**9**	… I thought about all the things that could go wrong.	☐	☐	☐	☐	☐
**10**	… my agitation made me unsure.	☐	☐	☐	☐	☐
**11**	… I felt well prepared.	☐	☐	☐	☐	☐

During the performance…

		1	2	3	4	5
**12**	… I could stop thinking about what might go wrong.	☐	☐	☐	☐	☐
**13**	… I could feel the agitation in my body.	☐	☐	☐	☐	☐
**14**	… I had fun and wanted to show what I could do.	☐	☐	☐	☐	☐
**15**	… I managed to control my agitation and stay calm.	☐	☐	☐	☐	☐
**16**	… I could concentrate on my musical performance.	☐	☐	☐	☐	☐
**17**	… I felt limited or disabled due to the way my body reacted to my agitation.	☐	☐	☐	☐	☐
**18**	… I had the feeling that my performance came across well.	☐	☐	☐	☐	☐
**19**	… my agitation made me unsure.	☐	☐	☐	☐	☐

During the performance… (continued)

	Not true at all 1	2	3	4	Very true 5
**20** … I knew exactly what I was doing.	☐	☐	☐	☐	☐
**21** … I felt inner strength.	☐	☐	☐	☐	☐
**22** … I could control my agitation.	☐	☐	☐	☐	☐

Now, after the performance...

	1	2	3	4	5
**23** … I think my performance came across well.	☐	☐	☐	☐	☐
**24** … it's hard to accept my performance as it was.	☐	☐	☐	☐	☐
**25** … I'm looking forward to my next performance.	☐	☐	☐	☐	☐
**26** … I accept my performance.	☐	☐	☐	☐	☐
**27** … I can switch off.	☐	☐	☐	☐	☐
**28** … it's hard to imagine that the audience liked my performance.	☐	☐	☐	☐	☐
**29** … I feel inner strength for the next performance.	☐	☐	☐	☐	☐
**30** … I am happy about my achievement.	☐	☐	☐	☐	☐
**31** … I feel stressed, unhappy.	☐	☐	☐	☐	☐
**32** … this performance proves that performing suits me.	☐	☐	☐	☐	☐
**33** … I'm mainly thinking about what didn't work.	☐	☐	☐	☐	☐

When considering the musical quality of my performance, I rate...

	terrible 1	pretty bad 2	less good 3	good 4	very good 5	excellent 6
**1** … the dynamic shaping as …	☐	☐	☐	☐	☐	☐
**2** … the rhythmic precision as …	☐	☐	☐	☐	☐	☐
**3** … the shaping of sound as …	☐	☐	☐	☐	☐	☐
**4** … the musical expression as …	☐	☐	☐	☐	☐	☐
**5** … the phrasing as …	☐	☐	☐	☐	☐	☐
**6** … the intonation as …	☐	☐	☐	☐	☐	☐
**7** … it in general as…	☐	☐	☐	☐	☐	☐
